# Rapid Differentiation of False Positives of Galactomannan Related to Contaminated Intravenous Fluids via a Pharmacokinetics Model and Innovative Web-Based Tool

**DOI:** 10.1093/ofid/ofaf088

**Published:** 2025-04-03

**Authors:** Raeseok Lee, Suein Choi, Hwajun Cha, Dukhee Nho, Sung-Yeon Cho, Seunghoon Han, Dong-Gun Lee

**Affiliations:** Division of Infectious Diseases, Department of Internal Medicine, College of Medicine, The Catholic University of Korea, Seoul, Republic of Korea; Vaccine Bio Research Institute, College of Medicine, The Catholic University of Korea, Seoul, Republic of Korea; Department of Clinical Pharmacology and Therapeutics, College of Medicine, The Catholic University of Korea, Seoul, Republic of Korea; Pharmacometrics Institute for Practical Education and Training, College of Medicine, The Catholic University of Korea, Seoul, Republic of Korea; Department of Clinical Pharmacology and Therapeutics, College of Medicine, The Catholic University of Korea, Seoul, Republic of Korea; Pharmacometrics Institute for Practical Education and Training, College of Medicine, The Catholic University of Korea, Seoul, Republic of Korea; Division of Infectious Diseases, Department of Internal Medicine, College of Medicine, The Catholic University of Korea, Seoul, Republic of Korea; Vaccine Bio Research Institute, College of Medicine, The Catholic University of Korea, Seoul, Republic of Korea; Division of Infectious Diseases, Department of Internal Medicine, College of Medicine, The Catholic University of Korea, Seoul, Republic of Korea; Vaccine Bio Research Institute, College of Medicine, The Catholic University of Korea, Seoul, Republic of Korea; Department of Clinical Pharmacology and Therapeutics, College of Medicine, The Catholic University of Korea, Seoul, Republic of Korea; Pharmacometrics Institute for Practical Education and Training, College of Medicine, The Catholic University of Korea, Seoul, Republic of Korea; Division of Infectious Diseases, Department of Internal Medicine, College of Medicine, The Catholic University of Korea, Seoul, Republic of Korea; Vaccine Bio Research Institute, College of Medicine, The Catholic University of Korea, Seoul, Republic of Korea

**Keywords:** aspergillosis, false-positive reactions, galactomannan, pharmacokinetics, web-based applications

## Abstract

This study developed a pharmacokinetic model and web-based tool to distinguish true invasive aspergillosis from false positives caused by contaminated fluids. By analyzing galactomannan kinetics, false positives were identified within 24 hours, improving diagnostic accuracy and aiding clinicians in early decision making while minimizing unnecessary interventions.

The *Aspergillus* antigen galactomannan (GM) assay is a key diagnostic tool for invasive aspergillosis (IA) due to the challenges of culturing *Aspergillus* and the limitations of histologic diagnostics [1, 2]. However, its diagnostic accuracy is often undermined by false-positive results, complicating clinical interpretation [3–5].

False positives have been identified from GM-contaminated fluids and successfully intervened by modifying the manufacturing process [3]. Yet, this problem persists, particularly with the ongoing use of mold in the production of fluids and β-lactam antibiotics, which significantly increase the risk of recurrent false positives [3, 6–8].

This study aimed to differentiate GM false positives from true IA by analyzing GM kinetics. We also developed and evaluated a software tool designed to assist clinicians in accurately interpreting GM results when false positives are suspected.

## METHODS

### Study Design and Setting

The case group (false-positive GM) included patients confirmed to have GM false positivity between July and August 2023 [3]. We selected patients whose GM index of contaminated fluid and subsequent changes in serum GM index were measured at 0 (pretest), 24, and 48 hours after discontinuing contaminated fluids. For GM kinetics modeling, a control group (true-positive GM) was included from patients diagnosed with proven or probable IA between January 2022 and June 2023, a period with a low probability of false positives [3]. Control cases were selected when the GM index was measured at least twice before antimold active agents were administered. Classification followed established criteria and was determined via consensus of 2 infectious disease specialists [1, 9].

The Platelia *Aspergillus* GM assay (Bio-Rad) was used to quantify GM index. The study protocol was approved by the institutional review board of Seoul St Mary's Hospital (KC23WISI0594), and the requirement for written informed consent was waived.

### Definitions of Covariates

We reviewed electronic medical records and collected covariates potentially affecting GM kinetics, including age, sex, glomerular filtration rate (GFR), and hepatic function based on the Child-Pugh classification [10]. In the model, GFR values >100 mL/min/1.73 m^2^ were capped at 100 mL/min/1.73 m^2^. Additionally, Child-Pugh class A and B were defined as mild to moderate hepatic dysfunction, whereas class C was defined as severe dysfunction.

### Population Pharmacokinetic Modeling

GM kinetics were analyzed by nonlinear mixed effects modeling, with all concentration-time data modeled simultaneously. The final model was selected according to the bayesian information criterion, maximizing the likelihood. Baseline characteristics (GFR, age, and major comorbidities) were evaluated as potential covariates. Detailed methods are described in the supplementary methods.

### Software Development

A web-based application was developed with R Shiny and the *mrgsolve* package to model the GM index profile based on the final pharmacokinetic model. The application was designed to predict false-positive GM results and recommend the minimum monitoring duration required to distinguish outcomes based on the initial GM index.

## RESULTS

### Baseline Patient Characteristics

Of 97 patients confirmed to have GM false positivity, 8 were selected for the case group. These patients had initial serum and fluid GM results, as well as follow-up GM assay results after contaminated fluids were discontinued. The control group consisted of 30 patients with proven or probable IA, each having a GM index measured at least twice prior to antifungal therapy. Patients in the case group were younger but had a higher initial GM index as compared with the control group (Table 1).

**Table 1.** Baseline Characteristics

	False Positive (n = 8)	True IA (n = 30)	Total (n = 38)	*P* Value
Age, y	42.5 (28.5–61.0)	62.0 (53.0–67.0)	61.0 (48.0–66.0)	.031
Sex: male	4 (50.0)	16 (53.3)	20 (52.6)	>.99
Diagnosis				.742
ALL	2 (25.0)	5 (16.7)	7 (18.4)	
AML	5 (62.5)	14 (46.7)	19 (50.0)	
Lymphoma	1 (12.5)	3 (10.0)	4 (10.5)	
MDS	0 (0.0)	1 (3.3)	1 (2.6)	
Myelofibrosis	0 (0.0)	1 (3.3)	1 (2.6)	
Multiple myeloma	0 (0.0)	6 (20.0)	6 (15.8)	
eGFR	133.5 (102.5–164.5)	136.0 (101.0–152.0)	136.0 (101.0–154.0)	.839
Severe hepatic dysfunction	0 (0.0)	0 (0.0)	0 (0.0)	
GM index				
Initial	2.6 (1.7–4.3)	1.6 (0.8–1.9)	1.6 (0.9–2.4)	.003
Follow-up	1.3 (0.4–2.3)^a^	2.5 (1.6–3.2)	2.3 (1.4–3.1)	.047
Time difference, h	24.0 (24.0–27.8)	71.7 (62.6–91.2)	71.1 (29.2–72.3)	.001

Values are presented as No. (%) or median (IQR).

Abbreviation: ALL, acute lymphoid leukemia; AML, acute myeloid leukemia; eGFR, estimated glomerular filtration rate; GM, galactomannan; IA, invasive aspergillosis; MDS, myelodysplastic syndrome.

^a^GM index was measured after the discontinuation of contaminated fluids.

### GM Pharmacokinetic Modeling

**Figure 1.**
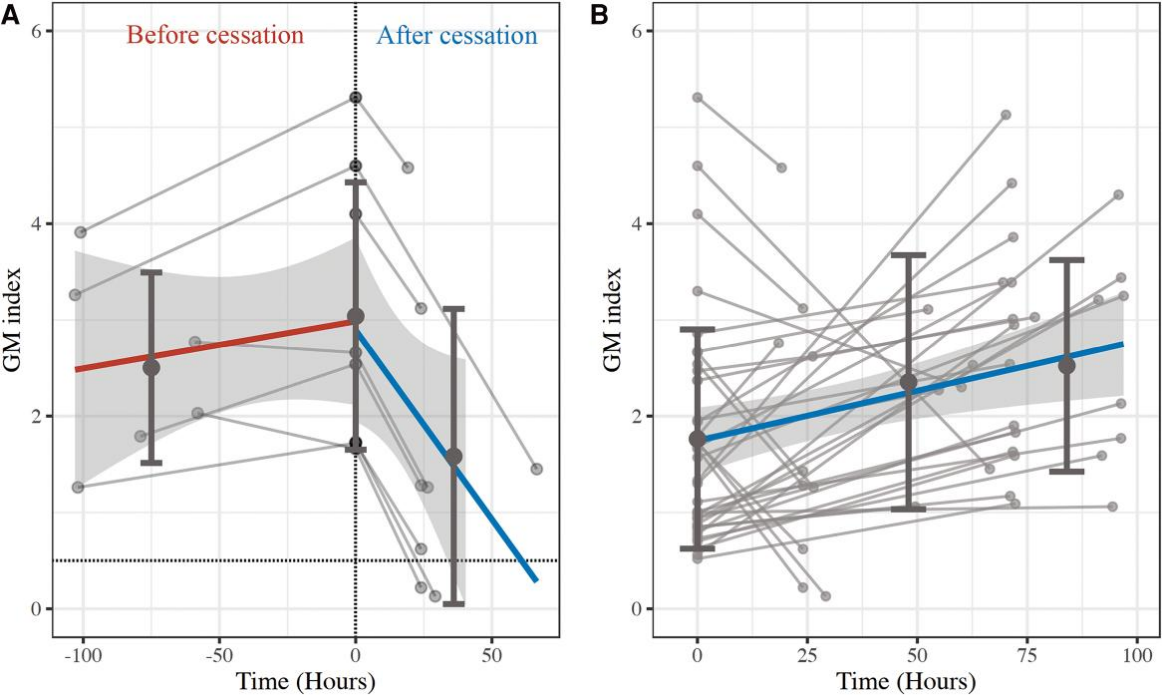
Time-concentration change of the galactomannan index: *A*, following the discontinuation of the galactomannan-contaminated solution; *B*, from the time when it turned positive until just before the initiation of antifungal therapy in patients with confirmed invasive aspergillosis. Solid line, median change; gray circle, individual observation; dashed line, 0.5 threshold for positive indices. Dark gray circle, mean value at each binned time frame; dark gray vertical line, ± standard deviation (SD) around the mean; gray shading, 95% CI interval of regression line. GM, galactomannan.

Following the discontinuation of contaminated fluids, GM index levels rapidly decreased in the false-positive group but significantly increased in the true IA group (*P* < .05; Table 1, Figure 1). The false-positive group had a higher initial GM index (median, 2.6; IQR, 1.7–4.3) as compared with the true IA group (median, 1.6; IQR, 0.8–1.9). GM concentrations in contaminated dextrose-containing fluids ranged from 8 to 4320 ng/mL, with a median 4.2 ng/mL (range, 3.2–6.6).

Pharmacokinetic modeling indicated a GM synthesis rate of 0.127 and an elimination rate constant of 0.030, with no significant covariates (Supplementary Table 1). The model predicted that, for an initial GM index of 3.0, levels would typically become negative (GM index <0.5) within a median of 2.3 days (95% CI, 1.51–3.8 days), with an estimated half-life of 23.6 hours (95% CI, 12.2–43.4 hours), indicating complete clearance within approximately 4.8 days. Simulations suggested that most false positives can be confirmed within 18 hours of stopping exposure, though lower initial GM indices require longer monitoring (Supplementary Table 2).

### Performance of Developed Web-Based Software

The developed web-based application, available at https://mychloe00.shinyapps.io/galactomannan/, accurately predicted the likelihood of true IA vs false positivity based on GM indices and the time intervals between measurements (area under the receiver operating characteristic curve, 0.93). The tool also recommended the optimal time to discontinue a suspected source of contamination (Supplementary Figures 1 and 2).

## DISCUSSION

This study demonstrated that GM false positives can be reliably distinguished from true IA cases within 24 hours by analyzing GM kinetics. The newly developed web-based tool provides clinicians with a practical, accessible means to simulate GM trends, significantly improving diagnostic accuracy for IA and reducing the risk of misinterpretation due to false positives.

False positives from GM contamination by *Aspergillus* used in carbohydrate fermentation have been consistently reported [3–8]. However, previous studies validating GM kinetics have primarily been conducted in animal models, with limited data available on human false-positive cases [10]. A previous study indicated a GM half-life of 2.4 days and a clearance time of 5.5 days after discontinuation of penicillin antibiotics [11]. Yet, as early diagnosis and treatment within 48.0 hours of infection are critical for therapeutic prognosis, waiting 5 days to determine false positivity is impractical [11].

Our findings show that the GM half-life in false-positive cases is much shorter—approximately 23.6 hours—allowing for false positivity to be differentiated within 18.0 hours. This early detection ability enables clinicians to diagnose IA accurately while minimizing the risk of misdiagnosing false positives. Since the GM index in true IA does not significantly decrease within 4 to 6 days of azole treatment, our model may offer a reliable approach for distinguishing false positives even up to a week after initiation of mold-active therapy [10]. However, consideration of parameters such as host factors, clinical findings, and radiologic features are crucial in diagnosis of IA.

The web-based software developed in this study is the first clinical application designed to distinguish false positives within 24 hours. This tool improves early diagnosis and therapeutic decision making for true IA while reducing unnecessary interventions triggered by false positives. Additionally, the tool detects contamination from other intravenous sources that exhibit similar GM elevation patterns, such as piperacillin/tazobactam [11]. In addition, even when GM patterns differ, clinicians can use the software to compare observed GM changes after discontinuing the suspected false-positive sources against simulated reference values, thereby enhancing their diagnostic decision making. Further research and modeling are needed to extend its application to false positivity arising from gastrointestinal absorption of contaminated materials. The software is freely available on the provided website.

Despite its strengths, this study has some limitations. First, the GM kinetics model could not be validated with external data due to a lack of additional suspected false-positive cases at our institution [3]. Second, the rate of GM increase varied by *Aspergillus* species. However, among the 30 true IA cases, only 3 were culture positive, and all were identified as *Aspergillus fumigatus*, thus preventing species-specific modeling [12]. Third, the applicability of the model to other sources of false positivity remains uncertain, and further validation studies are required for this. Finally, although we included a control group during the period of low probability of using contaminated fluids, the possibility of GM-contaminated dextrose fluid infused to the control group was not completely ruled out.

In conclusion, GM false positives can be effectively differentiated from true IA cases within 24 hours by analyzing GM kinetics after discontinuation of contaminated sources. The web-based tool developed from this model offers clinicians a valuable resource for accurately diagnosing IA and reducing false positives.

## Supplementary Material

ofaf088_Supplementary_Data

## Supplementary Data

Supplementary materials are available at *Open Forum Infectious Diseases* online. Consisting of data provided by the authors to benefit the reader, the posted materials are not copyedited and are the sole responsibility of the authors, so questions or comments should be addressed to the corresponding author.

## Notes

***Acknowledgments.*** We acknowledge the Department of Clinical Pharmacology for its consultation and statistical and technical support for this study.

***Author contributions.*** R. L., S. C., D. N., and D.-G. L. conceptualized and R. L., S. C., S.-Y. C., and S. H. coordinated this study. R. L., S. C., H. C., and D. N. performed data analysis and H. C., S.-Y. C., S. H., and D.-G. L. interpreted the data. R. L. and S. C. drafted manuscript, and all authors have fully reviewed the manuscript. All authors agree with all contents and conclusions of this manuscript.

***Data availability statement.*** The full data are available upon request from the corresponding author.
