# Supplementary material for: Rapid Differentiation of False Positives of Galactomannan Related to Contaminated Intravenous Fluids via a Pharmacokinetics Model and Innovative Web-Based Tool
Source: Open Forum Infect Dis. 2025 Apr 3;12(4):ofaf088. doi: 10.1093/ofid/ofaf088 (PMC11965789; doi:10.1093/ofid/ofaf088)
Supplement: ofaf088_Supplementary_Data [file ofaf088_supplementary_data.docx]

**Supplementary materials**

**Table of contents:**

[**Supplementary Method** 2](#_Toc175572987)

[**Supplementary Table 1.** Final population pharmacokinetic parameter 6](#_Toc175572988)

[**Supplementary Table 2.** Recommended stopping time of suspected false-positive source by observed galactomannan index 7](#_Toc175572989)

[**Supplementary Figure 1.** Web-based application for galactomannan pharmacokinetic simulation 8](#_Toc175572990)

[**Supplementary Figure 2.** Receiver operating characteristic curve of predicting the false-positive 9](#_Toc175572991)

[**Supplementary Figure 3.** Goodness-of-fit plots of the final population pharmacokinetic model 10](#_Toc175572992)

[**Supplementary Figure 4.** Prediction-corrected visual predictive check of the final model. 11](#_Toc175572993)

[**References** 12](#_Toc175572994)

# **Supplementary Method**

***Population pharmacokinetic modeling***

Galactomannan (GM) kinetics were analyzed using nonlinear mixed-effects modeling. All concentration-time data were modeled simultaneously, with the final model selected based on the Bayesian information criterion to maximize likelihood. Baseline characteristics, including glomerular filtration rate (GFR), age, sex, diagnosis, and Child-Pugh score, were evaluated as potential covariates. The model accounted for typical population values, as well as random effects such as interindividual variability (IIV) and residual variability.

***Population pharmacokinetic/pharmacodynamic model development***

Sequential population pharmacokinetic (PK) models were developed based on plasma concentrations of galactomannan (GM). Nonlinear mixed-effects modeling was conducted using NONMEM version 7.4 (Icon Development Solutions, Ellicott City, MD), with first-order conditional estimation methods and interactions applied for all model runs. Run management was performed with Pirana version 23.10.1 (Certara Inc., NJ). Prediction-corrected visual prediction checks (pcVPC), data manipulation, and visualization were carried out using R version 4.2.1 (Austrian Foundation for Statistical Computing, Vienna, Austria) with the lattice, xpose4, and ggplot2 packages. For PK analyses, concentrations below the limit of quantification (BQL) were assigned a value of 0, and final model parameter estimates were evaluated after excluding BQL data.

Structural models were selected based on established indirect-response GM models by assessing objective function values (OFVs), parameter estimate precision, diagnostic plots, and model stability [1]. Prior information from known GM models was used to support parameter estimation [1, 2]

Where GM antigen is assumed to be produced by a zero-order synthesis rate and degraded via a first-order process:

$$\frac{d\left( GM \right)}{dt}=k_{\text{in}}-k_{\text{out}}\left( GM \right)$$

Once the PK model met acceptance criteria, the population elimination-related PK parameter(k_out_) estimates were fixed, and the sequential analysis was performed for synthesis rate (k_in_) estimation. For the PK parameter, the following distribution is assumed:

$$P_{ij}=\theta_{\text{Pop},j}\times\exp\left( \eta_{ij} \right)$$

Where P_ij_ denotes the estimate for parameter *j* in the ith individual; θ_Pop,j_ is the population value for the parameter *j*; and η_ij_ denotes the deviation from average population value for parameter *j* in the *i*th individual with a mean of zero and variance of x^2^.

Initially, between-subject variability (BSV) was estimated for all parameters. Subsequently, η-shrinkage and model stability were evaluated to determine which BSV parameters to retain. The covariance between BSV parameters for clearance and volume of distribution was estimated, and a correlation coefficient was calculated. An additive residual error model was used throughout.

***Covariate analysis***

Covariates were evaluated for inclusion in the PK model using visual inspection methods. Box plots and scatter plots were used to explore the relationship between individual deviations from typical population values covariates (categorical and continuous, respectively). Covariates assessed included age, weight, sex, GFR, and Child-Pugh score. A forward inclusion (P < 0.05 and ΔOFV >3.8) and backward elimination (P < 0.01 and ΔOFV >6.6) approach was employed to determine the statistical significance of each covariate's inclusion in the model. Descriptive statistics of individual empirical Bayesian estimates were also calculated.

All continuous covariates were tested using a power model and centered using the median covariate value for the sample as follows:

$$P_{\mathrm{ij}} = \theta_{\text{Pop},j} \times\left( \frac{\text{cov}_{i}}{\text{cov}_{m}} \right)^{\theta_{\text{cov}}}$$

$$P_{\mathrm{ij}} = \theta_{\text{Pop},j} \times\theta_{\text{cov}}^{\text{cat}}$$

Where cov_i_ represents the individual covariate value; cov_m_ denotes the median covariate value of the population; θ_cov_ is a parameter that denotes the covariate effect; and *cat* is a categorical variable that takes a value between 0 and 1 for the categorical covariates (comedication administered, genotype status, and assisted status) analyzed. For example, the categorical variable takes 0 for males and 1 for females.

Standard diagnostic methods were used to evaluate model performance, including assessing successful minimization, diagnostic plots, plausibility and precision of parameter estimates, changes in the OFV, and shrinkage values. The precision of PK parameters was further assessed through nonparametric bootstrapping (500 replicates) to generate 95% confidence intervals for the parameter estimates.

# Prediction-corrected visual predictive checks were performed for the final model, generating 1,000 Monte Carlo simulation replicates per time point. Further details, including diagnostic plots and pcVPC plots, are provided in **Supplementary Figures 3 and 4.**

# **Supplementary Table 1.** Final population pharmacokinetic parameter

| **Parameter** | **Description** | **Value** | **RSE (%)** |
| --- | --- | --- | --- |
| **Fixed effect** |  |  |  |
| k_in_ (GM index/hr) | Synthesis rate | 0.127 | 22.21 |
| k_out_ (/hr) | Elimination rate constant | 0.030^a^ | 32.46 ^a^ |
| POP_max_ | Theoretical maximum GM concentration | 6.410 | 19.97 |
| **Interindividual variability** |  |  |  |
| ω_kin_ (CV%) | Interindividual variability of k_in_ | 0.326 (62.08%) | 32.0 |
| ω_kout_ (CV%) | Interindividual variability of k_out_ | 0.169 (42.91%) | 43.0 |
| **Residual error** |  |  |  |
| Additive (GM index) | Additive residual error | 0.409 | 22.83 |

^a^Priori estimated with false positive patient data

CV, Coefficient of variation; GM, galactomannan; RSE, Residual standard error.

# **Supplementary Table 2.** Recommended stopping time of suspected false-positive source by observed galactomannan index

| **Observed GM index** | **Recommended stopping time (hr)** | **Predicted time to reach LLOQ (hr)** | |
| --- | --- | --- | --- |
|  |  | **Median** | **95% CI** |
| 1 | 24 | 21.30 | 13.60 – 33.25 |
| 1.5 | 22 | 34.25 | 21.55 – 55.75 |
| 2 | 17 | 43.25 | 27.65 – 70.40 |
| 2.5 | 15 | 50.50 | 32.00 – 82.85 |
| 3 | 14 | 55.65 | 36.20 – 90.80 |
| 3.5 | 13 | 62.20 | 39.95 – 98.70 |
| 4 | 11 | 65.35 | 41.75 – 101.90 |

CI, confidence interval; GM, galactomannan; LLOQ, lower limit of quantification.


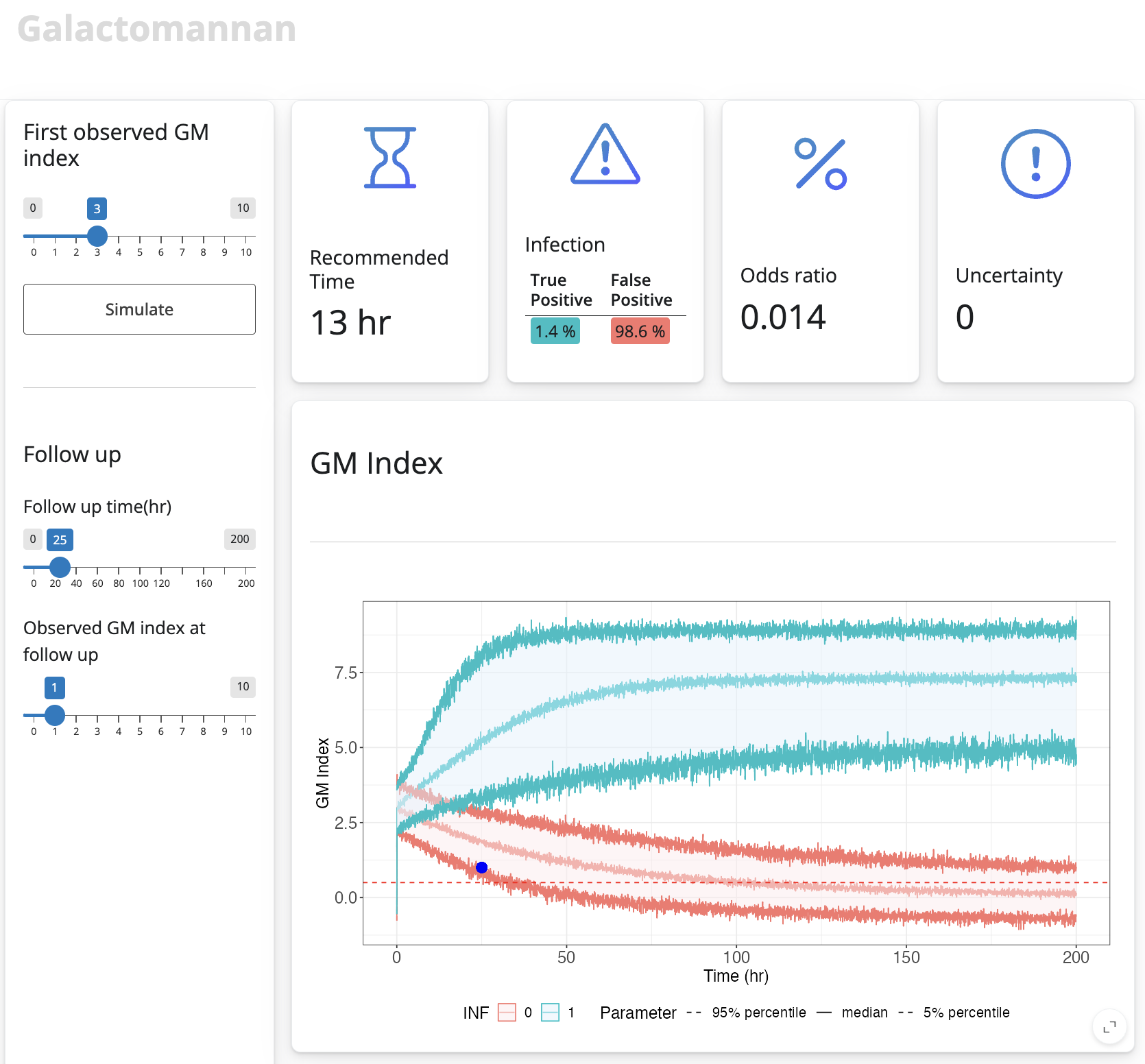


# **Supplementary Figure 1.** Web-based application for galactomannan pharmacokinetic simulation. It suggests recommending appropriate downtime for suspected false positive sources and predicts the likelihood of false positives. GM, galactomannan.

# **Supplementary Figure 2.** Receiver operating characteristic curve of predicting the false-positive. AUC, area under curve


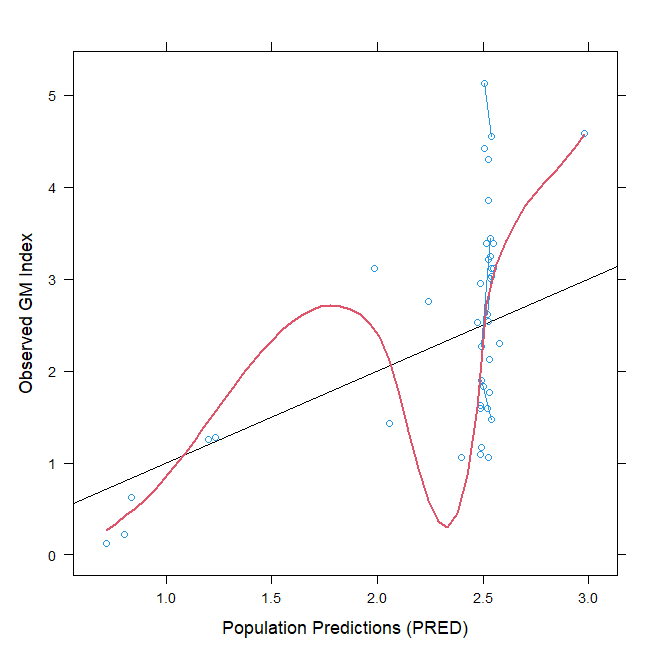

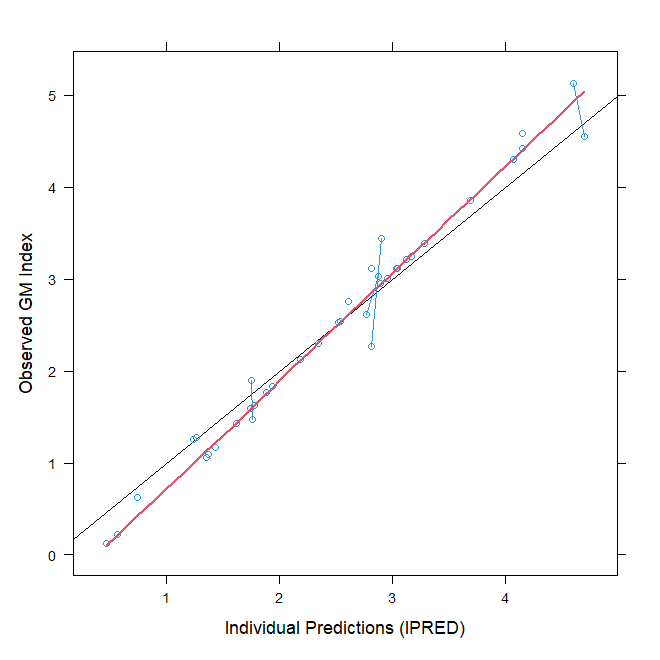

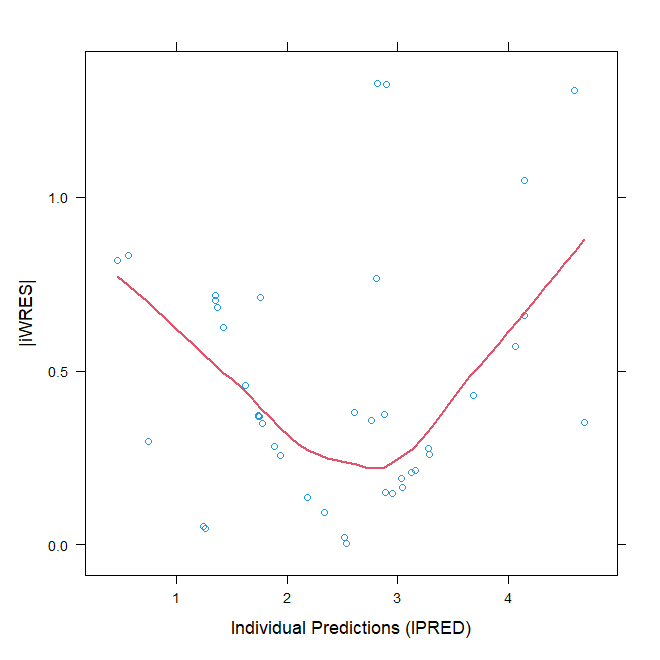

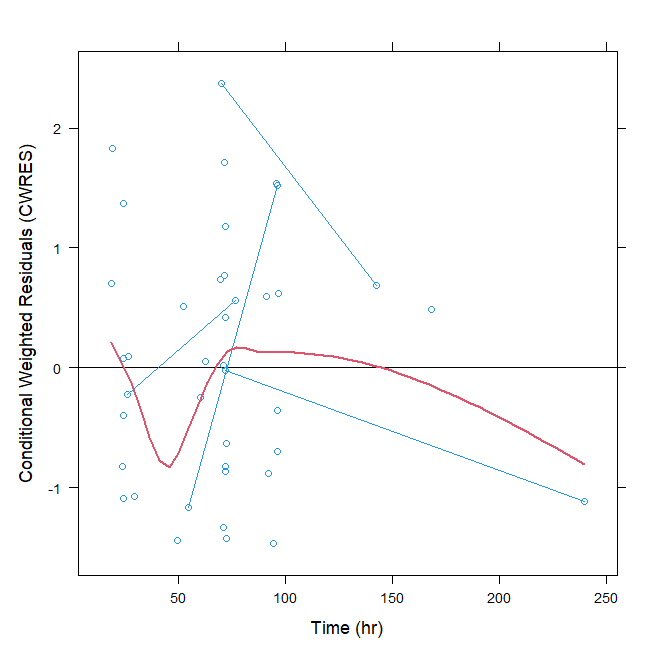


(A)

(C)

(D)

(B)

# **Supplementary Figure 3.** Goodness-of-fit plots of the final population pharmacokinetic model. Circles indicate the observed/predicted galactomannan index. The black lines indicate the identity or zero line. The red lines indicate the locally weighted scatterplot smoothing line. (**A**) Scatterplot of PRED versus observed GM index. (**B**) Scatterplot of IPRED versus observed GM index. (**C**) Scatterplot of IPRED vs. iWRES. (**D**) Scatterplot of Time vs. CWRES. PRED, population predictions; IPRED, individual predictions; iWRES, individual weighted residuals; CWRES, conditional weighted residuals

#
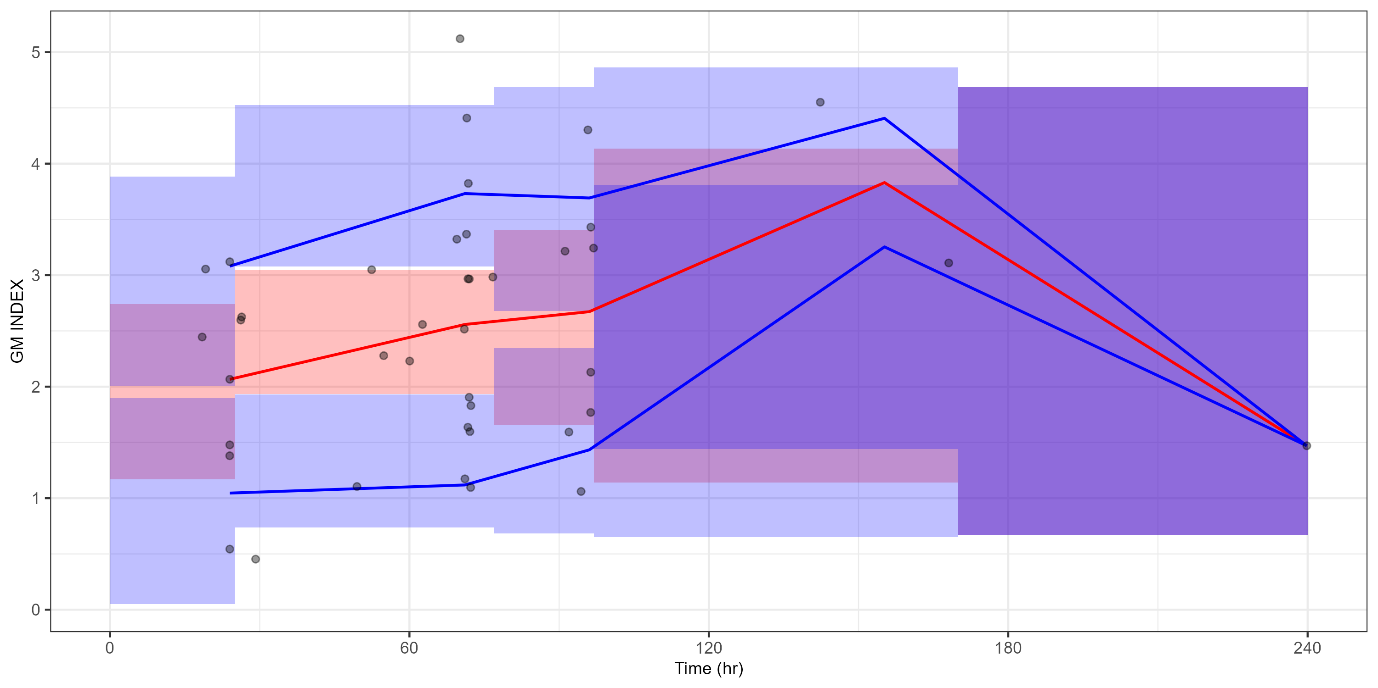
**Supplementary Figure 4.** Prediction-corrected visual predictive check of the final model. The solid red line represents the median of the prediction-corrected galactomannan index, and the semitransparent red field represents a simulation-based 95% confidence interval for the median. The observed 10% and 90% percentiles are presented with blue lines, and the 95% CIs for the corresponding simulation-based percentiles are presented as semitransparent blue fields. The prediction-corrected observed galactomannan index is presented as grey circles. GM, galactomannan.

# **References**

1. Aubry, R. Porcher, J. Bottero, et al. A. Sulahian, Occurrence and kinetics of false-positive Aspergillus galactomannan test results following treatment with beta-lactam antibiotics in patients with hematological disorders. Journal of Clinical Microbiology **2006**; 44(2): 389-94.
2. L.J. Huurneman, M. Neely, A. Veringa, et al. Pharmacodynamics of Voriconazole in Children: Further Steps along the Path to True Individualized Therapy. Antimicrobial Agents and Chemotherapy **2016**; 60(4):2336-42.
